# Driving-Related Glucose Monitoring Practices Among Insulin-Treated Adults With Type 2 Diabetes

**DOI:** 10.1177/19322968261450632

**Published:** 2026-05-30

**Authors:** Katharine Barnard-Kelly, Ryan Charles Kelly, Anthony Chen, Abdul Paracha, Pratik Choudhary, Tomas Walker, David Kerr

**Affiliations:** 1 BHR Limited, Fareham, UK; 2 Spotlight-AQ Limited, Portsmouth, UK; 3 Leicester Diabetes Centre, University of Leicester, UK; 4 Sutter Health Center for Health Systems Research, Santa Barbara, CA, USA

**Keywords:** driving, safety, type 2 diabetes, glucose monitoring

## Abstract

**Introduction:**

Driving requires complex cognitive, motor, and sensory coordination, all of which may be adversely affected by diabetes. For individuals treated with insulin, hypoglycemia represents the principal safety concern. Although many jurisdictions provide guidance for insulin-treated drivers, adherence to these recommendations remains unclear.

**Methods:**

An online survey was conducted among 500 licensed drivers with insulin-requiring type 2 diabetes (T2D) from the United States and the United Kingdom, not using continuous glucose monitoring (CGM). The survey included discrete and open-ended questions relating to diabetes management and driving behaviors. Descriptive and inferential statistical analyses were performed.

**Results:**

Participants were predominantly male (59%, n = 293) with a mean T2D duration of eight years. Insulin regimens included basal-only therapy (54%, n = 257), multiple daily injections (39%, n = 197), and continuous subcutaneous insulin infusion (9%, n = 46). Severe hypoglycemic event(s) in the preceding 12 months were reported by 34%, and 54% had evidence of impaired awareness of hypoglycemia (Clarke score >4). Furthermore, 37% (n = 185) limited their driving due to insulin-related concerns, and 72% (n = 359) worried particularly about hypoglycemia. Only 34% (n = 168) reported checking glucose levels before driving >75% of the time. If feeling hypo before driving, 59% (n = 295) would take a snack but drive immediately. If feeling hypoglycemic while driving, 10% (n = 48) would attempt to ‘get to their destination fast’. Only 27% (n = 138) carried fast-acting carbohydrates, and 42% (n = 210) felt safe to drive ‘as soon as I feel better’ while 32% (n = 163) would ‘wait 45 minutes and recheck their glucose levels’.

**Conclusions:**

Potentially unsafe driving behaviors are common among adults with insulin-treated T2D. There appears to be a need to improve the understanding of safe driving guidance.

## Introduction

Driving a motor vehicle safely requires coordination of a highly complex set of physiological, anatomical, and behavioral systems. Data varies on crash risk in individuals with diabetes, with studies conducted in the United States reporting significantly increased crash risk in drivers with diabetes when compared to drivers without diabetes (risk ratio = 1.284; 95% confidence interval [CI] = 1.124-1.466; *P* < .0001); however, studies conducted in non-US countries did not demonstrate such an increased risk (risk ratio = 1.035; 95% CI = 0.720-1.487; *P* = .854).^1^ As driving is a complex skill requiring visuospatial function, rapid information processing, vigilance, and sound judgment, hypoglycemia can result in cognitive impairment and psychomotor slowing, leading to functional deficits that may affect driving performance. Furthermore, driving itself imposes a metabolic demand on the brain that can, under certain circumstances, provoke hypoglycemia.^2^ A particular diabetes-related driving concern is sudden incapacitation due to hypoglycemia, including low glucose levels without warning symptoms.^3^

Individuals using insulin who drive are advised to monitor glucose prior to starting their journey and periodically during longer trips (in the United Kingdom, at least every 2 hours) due to the risk of hypoglycemia.^4^ Training on driving safety has also been recommended for motorists with diabetes following diagnosis,^5^ with key risk factors for driving impairment including previous episodes of severe hypoglycemia, prior hypoglycemia while driving, strict glycemic control (defined as low HbA1c), and absence of regular blood glucose monitoring.^5^ Specifically, in the United Kingdom, the Driver and Vehicle Licensing Agency (DVLA) recommends that insulin-treated drivers carry fast-acting carbohydrate and a blood glucose meter at all times. Drivers should measure glucose before driving and are advised to raise blood glucose if levels are between 4 and 5 mmol/L (<90 mg/dL) prior to driving.^6^ If hypoglycemia occurs (glucose <3.9 mmol/L [70 mg/dL]) while driving, drivers are advised to stop driving immediately and wait at least 45 minutes after recovery (glucose >5 mmol/L [90 mg/dL]) before resuming driving. Guidance from the American Diabetes Association^7^ similarly recommends that insulin-treated drivers carry a blood glucose meter, check glucose before and periodically during longer journeys, and keep readily available snacks containing carbohydrate, fat, and protein. Drivers are advised to stop safely if symptoms of hypoglycemia occur, treat low glucose appropriately, and not resume driving until blood glucose and cognition have fully recovered.^8^ Despite the availability of these recommendations, surveys suggest that a substantial proportion of drivers with type 1 diabetes continue to drive during hypoglycemia and may believe it is safe to drive at blood glucose levels below 70 mg/dL (3.9 mmol/L).^3^

The annual incidence of severe hypoglycemia among individuals with type 1 diabetes ranges between 30% and 40%. Adults with type 2 diabetes (T2D) requiring insulin treatment experience hypoglycemia less frequently; however, the risk of severe hypoglycemia increases with the duration of insulin therapy.^9^ Many jurisdictions provide driving-related guidance for individuals using insulin, but the extent to which these recommendations are communicated, understood, and followed remains unclear.

The aim of this study was to conduct an anonymized survey to determine the extent of adherence to driving safety guidelines among adults with T2D using insulin.

## Methods

An online survey was distributed between June and September 2025 to adults with T2D who were using insulin therapy and had previously stated they were willing to take part in research in the United Kingdom (n = 100) and the United States (n = 400). The survey is attached as Supplemental Appendix 1. The survey was written in UK English and translated into US English by native speakers on the research team. Adults with T2D using insulin and continuous glucose monitoring (CGM) were excluded because in the United Kingdom such devices are not routinely reimbursed amongst insulin using adults with T2D, and while this is not the case in the United Kingdom, CGM use was an exclusion criterion to maintain consistency across UK and US participant populations. Furthermore, there are currently no consensus guidelines for the application of CGM to improve driving safety.

The survey was designed by health care professionals (HCPs) and people with diabetes using an iterative co-design methodology. Informed consent was received prior to survey completion. Recruitment was conducted via a third-party agency, Sago, from their database of individuals with diabetes willing to take part in research. Screening questions provided multiple opportunities to ensure adults with type 1 diabetes were not able to enter and complete the survey. The survey contained n = 38 items including free text response questions, so that participants could provide further detail to their responses if desired (see Supplemental Appendix 2 for the survey). The survey was piloted with four adults with diabetes prior to use for acceptability with no revisions made to the final version. Descriptive and inferential statistical analyses were conducted using SPSS Statistics 22^10^ with content and thematic analyses conducted on the free text responses. Two researchers experienced in qualitative research methods analyzed the free text responses and conducted thematic and content analyses thereof. Descriptive analyses were conducted on the total sample and relevant subgroups, including gender, age, duration of diabetes, and duration of insulin therapy.

## Results

We received n = 500 completed survey responses (male n = 293, 59%). The median survey completion time was 8.5 minutes. The mean duration of insulin-treated diabetes was eight years (range = <1-35 years, SD = 7 years). All participants reported having a driving license and regularly driving a vehicle. Participant demographic and therapy information are presented in Table 1.

**Table 1.** Demographic and Therapy Data.

	Total		The United States		The United Kingdom		
	N	%	N	%	N	%	*P*-value
Gender							
Male	293	58.60	223	55.75	70	70.00	<.001
Female	207	41.40	177	44.25	30	30.00	
Age (years)							
17-25	3	0.60	1	0.25	2	2.00	<.001
26-35	27	5.40	9	2.25	18	18.00	
36-45	152	30.40	116	29.00	36	36.00	
46-55	110	22.00	93	23.25	17	17.00	
56-65	121	24.20	109	27.25	12	12.00	
66+	87	17.40	72	18.00	15	15.00	
Therapy							
Basal insulin	257	51.40	201	50.25	56	56.00	.040
Multiple daily injections of insulin	197	39.40	167	41.75	30	30.00	
Insulin pump	46	9.20	32	8.00	14	14.00	

Demographic and therapy information of drivers with T2D are presented in Table 1. There were more female drivers in the United States (n = 177, 44.25%) compared to the United Kingdom (n = 30, 30.00%) (*P*-value < .001). The US drivers were older (>45 years) (n = 274, 68.50%) compared to the UK drivers (n = 44, 44.00%) (*P*-value < .001). There were a higher number of US drivers with type 2 diabetes on multiple daily injections of insulin (n = 167, 41.75%) compared to UK drivers with type 2 diabetes (n = 30, 30.00%) (*P*-value = .040). The mean duration of type diabetes in the United States is 8.45 (SD = 7.10) and in the United Kingdom is 7.05 (SD = 6.41) (*P*-value = .076).

**Figure 1.**
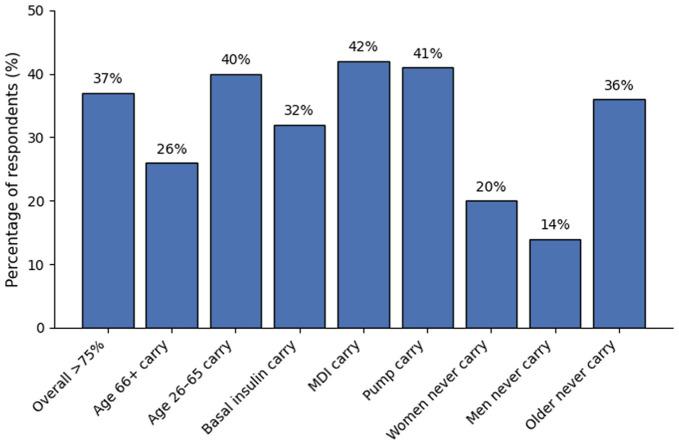
How often do you carry a blood glucose meter and strips when driving? Only 37% of respondents carry a blood glucose meter and strips more than 75% of the time. Those aged 26 to 65 years were more likely to do so than participants aged 66+ years; MDI users were more likely to do so than basal insulin or insulin pump users; and women were more likely to never to do so than men, with older participants more likely than younger participants to not carry blood glucose meters or strips.

The frequency of respondents reporting that they had access to a blood glucose meter and strips while driving varied by age and insulin delivery system (Figure 1).

The most commonly cited reasons for not carrying a blood glucose meter included forgetting to do so (n = 92), no perceived need to do so or never had an issue (n = 65), or taking short trips (n = 44).

Responses to survey items pertaining to the frequency of glucose checks prior to and during driving are presented in Table 2.

**Table 2.** Frequency of Blood Glucose Checks.

		Gender		Current age						Therapy		
	Total	Male	Female	17-25	26-35	36-45	46-55	56-65	66+	Basal insulin	MDI	Insulin pump
How often do you check your glucose before driving?												
>75% of the time	168 (34%)	96 (33%)	72 (35%)	1 (33%)	7 (26%)	51 (34%)	38 (35%)	42 (35%)	29 (33%)	73 (28%)	74 (38%)	21 (46%)
50-75% of the time	110 (22%)	71 (24%)	39 (19%)	2 (67%)	5 (19%)	38 (25%)	31 (28%)	26 (22%)	8 (9%)	58 (23%)	40 (20%)	12 (26%)
25-50% of the time	106 (21%)	67 (23%)	39 (19%)	0	11 (41%)	45 (30%)	18 (16%)	18 (15%)	14 (16%)	49 (19%)	49 (25%)	8 (17%)
< 25% of the time	72 (14%)	41 (14%)	31 (15%)	0	3 (11%)	15 (10%)	15 (10%)	15 (12%)	24 (28%)	47 (18%)	21 (11%)	4 (9%)
Never	44 (9%)	18 (6%)	26 (13%)	0	1 (4%)	3 (2%)	8 (7%)	20 (17%)	12 (14%)	30 (12%)	13 (7%)	1 (2%)
How often do you check your glucose when you are driving?												
Every 2 hours	143 (29%)	102 (35%)	41 (20%)	0	12 (44%)	52 (34%)	37 (34%)	23 (19%)	19 (22%)	51 (20%)	67 (34%)	25 (54%)
When I feel I’m going low	81 (16%)	51 (17%)	30 (15%)	1 (33%)	3 (11%)	10 (7%)	16 (15%)	25 (21%)	26 (30%)	47 (18%)	31 (16%)	3 (7%)
When I feel unwell	70 (14%)	28 (10%)	42 (20%)	0	2 (7%)	17 (11%)	18 (16%)	23 (19%)	10 (12%)	44 (17%)	22 (11%)	4 (9%)
At the start & the end of the journey	60 (12%)	34 (12%)	26 (13%)	1 (33%)	3 (11%)	22 (15%)	12 (11%)	17 (14%)	5 (6%)	34 (13%)	23 (12%)	3 (7%)
Only before setting off on my journey	92 (18%)	47 (16%)	45 (22%)	0	2 (7%)	28 (19%)	15 (15%)	25 (21%)	22 (25%)	57 (22%)	29 (15%)	6 (13%)

Other responses included every 60 to 90 minutes (n = 33), more than once an hour (n = 19), and when stuck in traffic (n = 2).

Rates of glucose checking were similar across all age ranges. The most commonly cited reasons for checking blood glucose levels while driving were feeling unwell and feeling as if hypoglycemia was occurring. Most participants also reported carrying fast-acting carbohydrate within easy reach in the vehicle while they were driving and carrying personal identification that included their use of insulin in case of injury in a road traffic accident. However, over a third of participants limited driving because they were living with insulin-treated diabetes (37%, n = 185), with this figure rising to more than half of participants aged 26 to 45 years (26-35 years: 52%, n = 14; 36-45 years: 57%, n = 86; 56-65 years: 17%, n = 21; and 66+ years: 17%, n = 15). Participants using intensive insulin therapies were more likely to limit driving. Specifically, 48% (n = 22) of the participants on insulin pump therapy limited driving compared to 42% (n = 82) of the participants on multiple daily injection (MDI) and 32% (n = 81) of the participants using basal insulin alone. Furthermore, men were more likely to limit driving than women (42% [n = 123] vs 30% [n = 62]).

Participants overwhelmingly answered ‘yes’ (95%) to the question if they had ever felt hypoglycemic while driving. However, concerning practices were reported in response to the question of ‘If I feel a hypo developing whilst I am driving, I . . .’. Almost 10% (n = 48) of participants reported that they would try to ‘get to their destination fast’. A further 35% (n = 173) reported stopping, checking with a glucose meter, treating and starting again, while another 28% (n = 138) reported that they had ‘fast acting carbs whilst driving’. Only 28% (n = 141) described best practice, namely stopping the vehicle, treating the low glucose level, and waiting 45 minutes after recovery before restarting their journey. There was similarly mixed data around understanding of the existing guidelines around when it is safe to drive following a hypoglycemic event. Overall, 42% (n = 210) of participants reported they would be safe to drive again as soon as they felt better. All other participants answered either 45 minutes later (33%, n = 163) or an hour later (25%, n = 127). Most were also worried about having a hypo while driving (a little worried: n = 292, 58%) or (very worried: n = 67, 13%). In response to the question about whether they had been involved in a car accident in the last five years, the vast majority (85%) reported they had not. Of those who reported having a crash, only 1.4% said it was related to hypoglycemia.

Hypoglycemia unawareness was common (see Table 3) with less than half of participants saying they always have warning symptoms of an impending low glucose level (46%, n = 231), but 22% (n = 231) could rarely or never tell low blood sugar by their symptoms. Objective assessment of hypoglycemia unawareness using the Clarke Score cut-off of ≥4 showed over half of participants had impaired hypoglycemia awareness (55%, n = 273), with men more affected than women (60%, n = 177 vs 46%, n = 96, respectively). A further 24% (n = 118) of participants had intermediate or impaired hypoglycemia awareness. Only 22% (n = 109) of participants reported normal hypoglycemia awareness (Table 3). Most participants said they would check their glucose more if they had access to CGM.

**Table 3.** Clarke Hypoglycemia Awareness Survey.

		Gender		Current age						Therapy		
	Total	Male	Female	17-25	26-35	36-45	46-55	56-65	66+	Basal insulin	MDI	Insulin pump
Impaired (≥4 R)	273	177	96	3	21	111	64	46	28	129	111	33
	54.60%	60.40%	46.40%	100.00%	77.80%	73.00%	58.20%	38.00%	32.20%	50.20%	56.30%	71.70%
Borderline (3 R)	118	59	59	0	4	21	32	35	26	64	46	8
	23.60%	20.10%	28.50%	-	14.80%	13.80%	29.10%	28.90%	29.90%	24.90%	23.40%	17.40%
Normal (≤2 R)	109	57	52	0	2	20	14	40	33	64	40	5
	21.80%	19.50%	25.10%	-	7.40%	13.20%	12.70%	33.10%	37.90%	24.90%	20.30%	10.90%

Comparisons between UK (n = 100) and US participants (n = 400) showed the UK cohort was younger (age <46, 56% vs 30% for US participants, *P* < .001) and more likely to be using basal insulin alone (57% vs 51%; *P* = .04) with the remainder using MDIs or insulin pumps. More UK drivers reported a history of severe hypoglycemic events and impaired hypoglycemic awareness than US drivers; however, this was not statistically significant (57% vs 46%, *P* = .075). The UK drivers were more likely to stop if they felt hypoglycemic and treat, waiting 45 minutes after recovery before starting (46% vs 24%, *P* < .001), whereas US drivers were more likely to have carbohydrates and continue driving (31% vs 15%, *P* < .001). The UK drivers were more likely to run higher glucose while driving (53% vs 33%, *P* < .001), despite similar levels of concern about hypoglycemia. More than half of drivers from both countries reported not carrying glucose meters and strips (the United Kingdom 61% vs the United States 62%).

## Discussion

For insulin-treated drivers, there is an established risk of developing impaired driving if hypoglycemia develops. This survey of driving-related glucose monitoring practices among 500 insulin-treated adults with T2D in the United States and the United Kingdom demonstrated a concerning lack of understanding of common guidelines and a number of concerning practices. Participants reported high rates of hypoglycemia unawareness, as well as failure to carry or use glucose monitoring before or during driving and inappropriate responses to hypoglycemia while driving. Almost 80% of participants reported borderline (24%) or impaired (55%) hypoglycemia awareness as assessed on the Clarke scale. Men were more likely to have impaired hypoglycemia awareness than women (60% vs 46%), with younger and middle-aged drivers experiencing more impaired hypoglycemia than drivers aged 55 years and over. This compares to data from Feher et al,^11^ who surveyed 1569 drivers and found 62% of participants reported hypoglycemic symptoms within the previous year. They further report that commuters and vocational drivers had higher rates of self-reported ‘poor self-management behavior’ compared with social drivers. Such behaviors included skipping meals, inconsistent medication use, and not checking blood glucose before driving.^11^ Unfortunately, we did not collect data on the nature of driving, but we would recommend this be included in future research.

Those on more intensive insulin regimens, ie, MDI or insulin pump therapy, were more likely to limit driving because of their diabetes. With more than a third of participants reporting they limit driving because they have insulin-dependent diabetes, despite low numbers across cohorts, those in younger and middle ages (aged 17-55) were more likely to do so than participants aged 56 years or above. While reasons for doing so are not provided, it could be that younger drivers are more exposed to more dangerous, frequent, and longer driving requirements, eg, for work, for family responsibilities, and during busier periods on the roads, as compared to older drivers who tend to take shorter journeys during lower-risk conditions with more stable routines.^12^ It could also be that older adults tend to have more stable blood glucose patterns, which could reduce the risk of hypoglycemia and subsequently impacted driving-related behaviors.^13^ These patterns warrant further investigation in future research, as they could also reflect increased anxiety or poor understanding of the rules, both of which are modifiable.

Driving for most people is associated with work or family obligations, eg, taking children to school, community to work, etc. As such, it is necessary to balance these lived experience priorities against the time it takes to treat hypoglycemia, particularly if a person feels safe to drive. Previous research has demonstrated that drivers often modify or selectively apply safety recommendations when driving is routine or necessary,^12^ as well as poor adherence to hypoglycemia avoidance behaviors by commuters and vocational drivers,^11^ reflecting time pressure and work demands rather than a lack of knowledge. Participant quotes in that research reflect the dilemma between ‘doing diabetes well’ competing with ‘doing life well’. The trade-offs drivers are making between safety and lived experience reflect a dangerous gamble when getting into a car. These findings raise concerns around the availability and/or quality of education related to driving and insulin. American Diabetes Association (ADA) Standards of Care emphasize that health care providers have a primary role in educating and counseling patients about driving safety when there is a risk of hypoglycemia, including for those on insulin.^14^ Specifically, clinicians should be knowledgeable about the risk of driving with diabetes and should discuss driving safety with patients at risk. Education should cover strategies to avoid and respond to hypoglycemia, understand individual risk, and know when it is safe or unsafe to drive including careful glucose monitoring and actions to take if levels are low.

The ADA also recognize the responsibility of the individual with diabetes for self-management, including safe driving practices informed by that education but don’t specify timepoints or frequency of glucose checks. In the United Kingdom, the DVLA does not require prescribers to guarantee that a patient fully understands the driving rules; rather, that is the driver’s legal responsibility. Adults with diabetes are expected to ‘know and comply with the legal rules’.^14^ Clinicians are required to advise about risks of hypoglycemia, remind patients of DVLA notification duties, consider driving impact when making treatment decisions, and document the advice given. There are also expectations for clinicians to advise patients not to drive if they have serious concerns, especially in the case of impaired awareness of hypoglycemia.^15^

The strengths of this study are the in-depth exploration of behaviors associated with driving among this population and the anonymity in the data collection. The examination of driving practices, alongside experiences and awareness of hypoglycemia, is novel and important. The study is limited by the self-selecting nature of the participant population, all of whom had signed up for research about diabetes. This creates a potential for bias that they may be more informed about diabetes care than people who did not sign up for such diabetes-related research. A further limitation was that we did not ask participants about whether they had informed driving regulatory bodies, such as the DVLA in the United Kingdom, about their insulin dependence, with notification being a requirement for driving in the United Kingdom. Such disclosure, however, comes with increased limitations on driving and, often, increased associated costs for insurance. The consequences of hypoglycemia unawareness, if disclosed, are that a driving license will be revoked for a minimum of 12 months. This, clearly, can have serious consequences for people to live their daily lives in terms of their ability to work, to get their children to school, to go to the grocery store, and so on. Perhaps, equally significantly, however, is the impact on people’s freedoms and their ability to live an autonomous life in the way they may have done previously.

In the United Kingdom, laws were recently changed that allowed drivers of both group 1 (personal vehicles) and group 2 (>3.5 tons or >8 passengers) to use CGM for glucose monitoring for driving. This change is set to make the roads safer, helping drivers to reduce the risk of hypoglycemia without running glucose levels higher than target and negatively impacting health. In the United Kingdom, all individuals with type 1 diabetes are entitled to use CGM systems, but access to CGM for those with insulin-treated T2D is very variable based on health care provider bias and local funding arrangements.^3^

## Conclusion

In conclusion, these data highlight the high prevalence of potentially unsafe driving-related behaviors among adults with insulin-requiring T2D. There is an urgent need for improved education of drivers and HCPs, as well as improved access to CGM for drivers to improve road safety for the traveling public.

## Supplemental Material

sj-docx-1-dst-10.1177_19322968261450632 – Supplemental material for Driving-Related Glucose Monitoring Practices Among Insulin-Treated Adults With Type 2 Diabetes Supplemental material, sj-docx-1-dst-10.1177_19322968261450632 for Driving-Related Glucose Monitoring Practices Among Insulin-Treated Adults With Type 2 Diabetes by Katharine Barnard-Kelly, Ryan Charles Kelly, Anthony Chen, Abdul Paracha, Pratik Choudhary, Tomas Walker and David Kerr in Journal of Diabetes Science and Technology
