# Supplementary material for: Driving-Related Glucose Monitoring Practices Among Insulin-Treated Adults With Type 2 Diabetes
Source: J Diabetes Sci Technol. 2026 May 30:19322968261450632. Online ahead of print. doi: 10.1177/19322968261450632 (PMC13223070; doi:10.1177/19322968261450632)
Supplement: sj-docx-1-dst-10.1177_19322968261450632 – Supplemental material for Driving-Related Glucose Monitoring Practices Among Insulin-Treated Adults With Type 2 Diabetes [file sj-docx-1-dst-10.1177_19322968261450632.docx]

**Appendix One: Driving Regulations / Guidelines for Adults with Diabetes Using Insulin (T1D and T2D)**

The UK guidelines ([inf294-a-guide-to-insulin-treated-diabetes-and-driving.pdf](https://assets.publishing.service.gov.uk/media/5d8b92dc40f0b6098d33fefe/inf294-a-guide-to-insulin-treated-diabetes-and-driving.pdf)) state that drivers with insulin treated diabetes are advised to take the following precautions:

 • You should always carry your glucose meter and blood glucose strips with you, even if you use a real time glucose monitoring system (RT-CGM) or flash glucose monitoring system (FGM).

 • You should check your glucose less than 2 hours before the start of the first journey and every 2 hours after driving has started.

 • A maximum of 2 hours should pass between the pre-driving glucose check and the first glucose check after driving has started.

 • More frequent testing may be required if for any reason there is a greater risk of hypoglycaemia for example after physical activity or an altered meal routine.

 • In each case if your glucose is 5.0mmol/L or less, eat a snack. If it is less than 4.0mmol/L or you feel hypoglycaemic do not drive

• Always keep an emergency supply of fast-acting carbohydrate such as glucose tablets or sweets within easy reach in the vehicle.

 • You should carry personal identification to show that you have diabetes in case of injury in a road traffic accident.

 • You should take extra care during changes of insulin regimens, changes of lifestyle, exercise, travel and pregnancy.

 • You must eat regular meals and snacks and take rest periods on long journeys. Always avoid alcohol.

Managing hypoglycaemia at times relevant to driving:

 • In each case if your glucose is 5.0mmol/L or less, eat a snack. If it is less than 4.0mmol/L or you feel hypoglycaemic do not drive.

 • If hypoglycaemia develops while driving stop the vehicle safely as soon as possible.

 • You should switch off the engine, remove the keys from the ignition and move from the driver’s seat.

 • You should not start driving again until 45 minutes after finger prick glucose has returned to normal (at least 5.0mmol/L). It takes up to 45 minutes for the brain to recover fully.

In the United States, each state has its own special licensing rules regarding medical conditions concerning diabetes. Some states implement these rules to all drivers with diabetes while others only apply them to diabetes individuals who take various medications such as insulin or exhibit symptoms such as

- vision problems (such as retinopathy)
- loss of consciousness
- seizures
- low blood sugar (hypoglycemia)
- foot problems (such as neuropathy)

The main medical concerns associated with diabetes that could affect driving safety are

*1*) hypoglycemia that impairs the ability to drive,

*2*) neuropathy that reduces the capacity to feel or operate foot pedals, and

*3*) retinopathy, cataracts, and/or glaucoma that impair the vision required for driving

Individuals with type 2 diabetes who are prescribed medications that stimulate insulin secretion, such as sulfonylureas, could face an increased risk of hypoglycemia episodes significant enough to affect driving safety.

US guidelines state ([Diabetes and Driving: A Statement of the American Diabetes Association | Diabetes Care | American Diabetes Association](https://diabetesjournals.org/care/article/47/11/1889/157262/Diabetes-and-Driving-A-Statement-of-the-American)):

- Always carry a blood glucose meter, a quick-acting source of glucose, and snacks with complex carbohydrate, fat, and protein in their vehicle
- Measure blood glucose before and periodically during longer drives
- Raise blood glucose if the level is <90 mg/dL before driving to prevent mild symptoms of hypoglycemia
- Safely stop the vehicle as soon as symptoms of low blood glucose are experienced and measure and treat the blood glucose level as needed
- Be ready to self-treat low blood glucose with fast-acting glucose, even in mild hypoglycemia ranges
- Not resume driving until blood glucose and cognition have recovered
- Work with a diabetes health care professional to consider and implement new diabetes management technologies (CGM, closed-loop insulin delivery system, etc.) where clinically indicated
- Use in-vehicle hand controls if experiencing loss of feeling in the foot
